# Incidence, influencing factors, and prognostic impact of intraoperative massive blood loss in adolescents with neuromuscular scoliosis: A STROBE-compliant retrospective observational analysis

**DOI:** 10.1097/MD.0000000000006292

**Published:** 2017-03-24

**Authors:** Rui Jia, Na Li, Bi-Yun Xu, Wei Zhang, Xiao-ping Gu, Zheng-Liang Ma

**Affiliations:** a Department of Anesthesiology; b Department of Statistics, Drum Tower Hospital, Medical School of Nanjing University, Nanjing, Jiangsu, China.

**Keywords:** adolescent, influencing factors, massive blood loss, neuromuscular, scoliosis

## Abstract

Factors influencing massive blood loss for neuromuscular scoliosis (NMS) patients.

Despite advances in surgical and anesthetic techniques, scoliosis surgery is still associated with intraoperative massive blood loss, which can result in postoperative mortality and morbidity. The aim of this study was to assess the incidence, influencing factors, and prognostic impact of intraoperative massive blood loss in adolescents with NMS.

A retrospective review of adolescents who underwent posterior spinal instrumentation and fusion for NMS was performed. Perioperative variables and data were recorded. Massive blood loss was defined as an estimated blood loss that exceeds 30% of total blood volume.

We obtained data for 114 patients, of whom 63 (55%) had intraoperative massive blood loss. Compared with those without, patients with massive blood loss were more likely to be older, have lower body mass indexes (BMIs), larger Cobb angles, more fused levels, more osteotomy procedures, and prolonged duration of operation. Logistic regression analysis identified the number of fused levels to be more than 12 (*P* = 0.003, odds ratio = 6.614, 95% confidence interval [CI]: 1.891–23.131), BMI lower than 16.8 kg/m^2^ (*P* = 0.025, odds ratio = 3.293, 95% CI: 1.159–9.357), age greater than 15 years (*P* = 0.014, odds ratio = 3.505, 95% CI: 1.259–9.761), and duration of operation longer than 4.4 hours (*P* = 0.016, odds ratio = 3.746, 95% CI: 1.428–9.822) as influencing factors. Patients with massive blood loss are associated with more intraoperative colloids infusion and blood transfusions (red blood cell and fresh frozen plasma), as well as postoperative drainage volume.

In adolescents with NMS who underwent posterior spinal instrumentation and fusion operations, intraoperative massive blood loss is common. The number of fused levels, BMI, age, and duration of operation are factors influencing intraoperative massive blood loss.

## 1 Introduction

Perioperative management of patients undergoing scoliosis surgery is technically challenging for both surgeons and anesthesiologists. Although surgical and anesthetic techniques have advanced substantially during the past decades,^[1–3]^ scoliosis surgery is still associated with intraoperative massive blood loss, and many patients often need blood product transfusion.^[4,5]^ However, neither intraoperative massive blood loss nor blood product transfusion is without risks.^[6,7]^ Both may adversely affect postoperative outcomes, increase health care costs, and prolong hospitalization.^[8]^ Therefore, identifying factors influencing intraoperative massive blood loss in patients undergoing scoliosis surgery is vital to establish an optimal blood conservation strategy in an era of blood supply shortage.

To date, only a few studies have elucidated factors influencing blood loss in patients undergoing scoliosis surgery.^[4,9–11]^ As the prevalence of neuromuscular scoliosis (NMS) is low, the sample sizes in these studies were relatively small. In that case, there is still an urgent need for further studies that will identify factors influencing blood loss, particularly intraoperative massive blood loss, in patients undergoing scoliosis surgery. Besides, the consequences associated with intraoperative massive blood loss have not been well established. Therefore, the present study was designed to assess the incidence, influencing factors, and prognostic impact of intraoperative massive blood loss in adolescents with NMS who underwent posterior spinal instrumentation and fusion (PSIF) operations.

## 2 Methods

### 2.1 Participants

**Figure 1:**
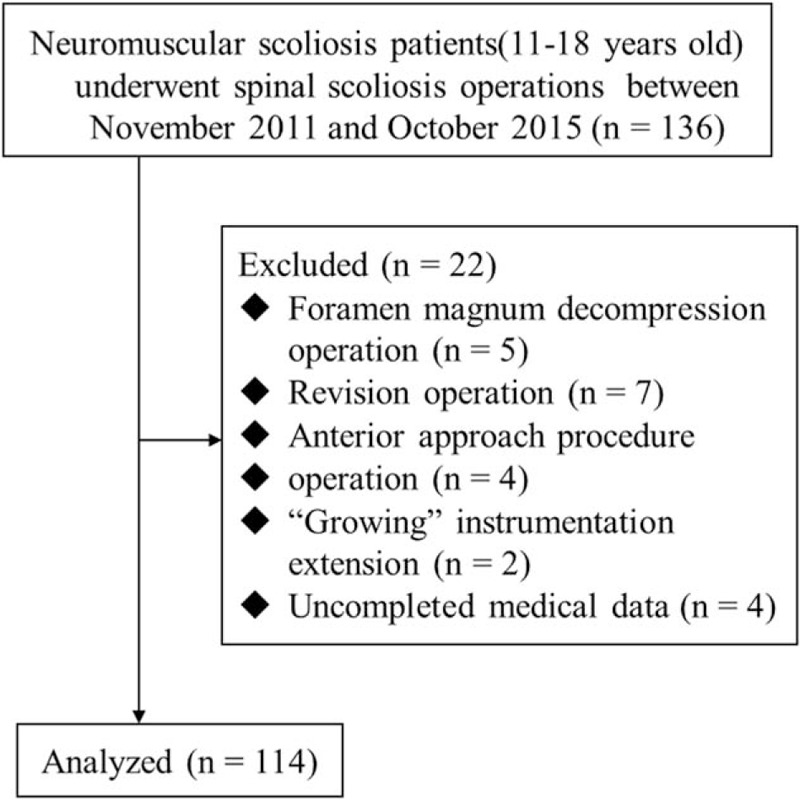
Patient screening and exclusion process.

This retrospective study was approved by the Institutional Review Board of Drum Tower Hospital, Medical School of Nanjing University, with reference number 2016-067-01. By means of an electronic medical records retrieval system, a total of 136 NMS patients, who met the inclusion criteria and received scoliosis surgery in Drum Tower Hospital, from November 2011 to October 2015, were retrospectively reviewed. Patients 11 to 18 years old with NMS were defined by 2 or more experienced senior attending doctors independently. From this group of 136 patients, we excluded patients who had a foramen magnum decompression operation (n = 5), revision operation (n = 7), anterior approach procedure operation (n = 4), “growing” instrumentation extension (n = 2), or uncompleted medical data (n = 4). The remaining 114 patients were included in the current research (Fig. 1).

### 2.2 Preoperative variables

A total of 114 NMS patients who received spinal correction and fusion surgeries were investigated in this study. Total intravenous anesthesia and posterior-only approach and pedicle screw fixation were applied to all cases. Preoperative data included gender, age, height, weight, American Society of Anesthesiologists grade, preoperative blood routine examinations, and coagulation function tests. Major curve Cobb angles and the extent of the vertebrae that needed to be fused were measured by operative surgeons according to preoperative full spine X-ray radiographs. Intraoperative variables included intraoperative estimated blood loss (EBL), duration of operation, data of transfusion, and fluid therapy. Postoperative data collection included the total blood volume (TBV) of postoperative drainage, the information of transfusion, and the length of hospital stay (LOS) after the operation. Calculated variables included corrected height (log Y = 0.011X − 0.177, where Y is the loss of trunk height due to the deformed spine and X is the greatest Cobb angle of the primary curve),^[12]^ body mass index (BMI, calculated by dividing weight in kilograms by corrected height in meters squared), implant density (divided implants by the number of available implant sites within the major Cobb angle),^[13]^ and TBV based on Nadler formula.^[14]^

The definition of massive blood loss was EBL/TBV ≥ 30%.^[4]^ According to this cut-off point, the patients were retrospectively divided into 2 groups: 1 group of massive blood loss (EBL/TBV ≥ 30%) and 1 group of minor blood loss (EBL/TBV < 30%).

### 2.3 Statistical analysis

Continuous variables were presented as mean ± SD or median (25th, 75th) percentiles and analyzed using Student *t* test or Mann–Whitney *U* test where appropriate, depending on the data distribution characteristics. Categorical variables were presented as frequencies (percentage) and analyzed using the chi-square test. Statistically significant continuous variables were transformed to categorical variables by their cut-off points, which were defined by receiver operating characteristic curve analysis. Forward stepwise binary logistic regression analysis was used to assess certain variables that showed statistical significance. The software package, Statistical Package for Social Sciences (SPSS, Chicago, IL; version 18.0) was used for all statistical analyses. Statistical significance was defined as *P* values less than 0.05.

## 3 Results

### 3.1 Descriptive summary of clinical variables

A total of 114 NMS PSIF medical cases were analyzed, of which 63 (55%) had intraoperative massive blood loss (EBL/TBV ≥ 30%). There was no statistical significance in TBV between the 2 groups (*P* = 0.388), although the intraoperative EBL in group-EBL/TBV ≥ 30% was larger than the other group (*P* < 0.001).

Compared to group-EBL/TBV < 30%, patients in group-EBL/TBV ≥ 30% were older, had lower BMIs, larger preoperative Cobb angles, prolonged duration of operation, given more vertebral levels of fusion, and osteotomy procedure (*P* = 0.010, *P* = 0.004, *P* < 0.001, *P* < 0.001, *P* = 0.001, and *P* = 0.013, respectively). The related clinical information is presented in Table 1.

**Table 1:**
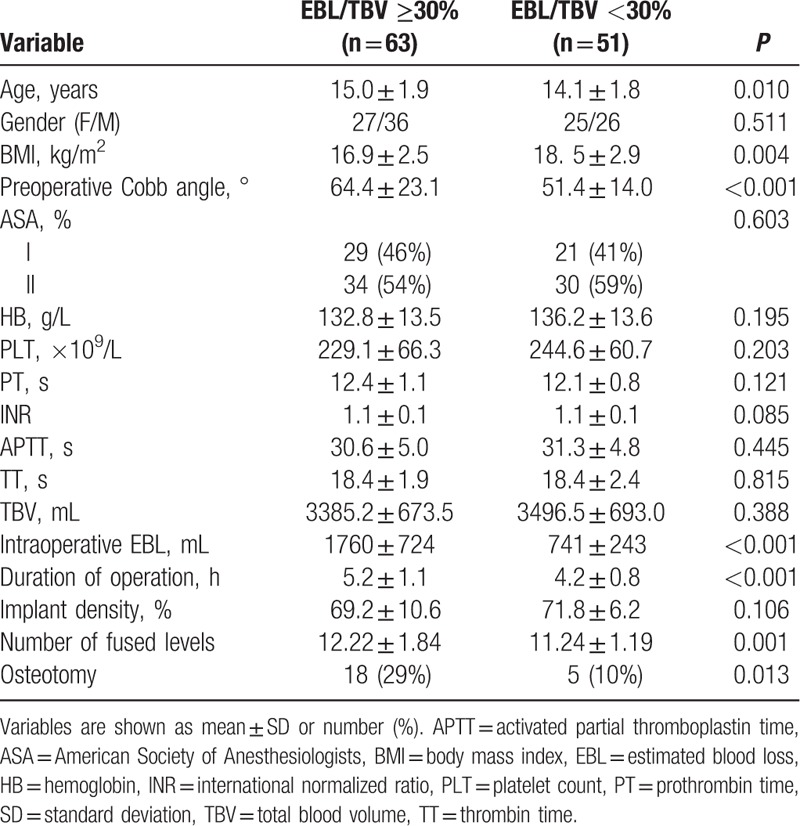
Patient characteristics.

### 3.2 Transformed variables and binary logistic regression analysis

For continuous variables that had significant differences between the 2 groups, we transformed these variables to categorical variables according to cut-off points, which were defined as the maximum value of the Youden index = sensitivity + specificity during the receiver-operating characteristic curve analysis. Through this method, clinical cut-off points, respectively, were 15 years old, 65° Cobb angle, BMI of 16.8 kg/m^2^, 12 fused levels, and 4.4 hours duration of operation.

For identifying the risk factors of massive blood loss, binary logistic regression analysis of covariances was used with osteotomy and aforementioned clinical cut-off points as the covariates. During the binary logistic regression analysis, 0.05 and 0.10 were defined as the entry and removal of the probability stepwise, respectively. Osteotomy and Cobb angle lost statistical significance in this logistic analysis. The equation of this logistic regression demonstrated that more than 12 fused levels, BMI lower than 16.8 kg/m^2^, age greater than 15 years, and duration of operation longer than 4.4 hours were the risk factors of massive blood loss. The details of binary logistic regression analysis are illustrated in Table 2.

**Table 2:**
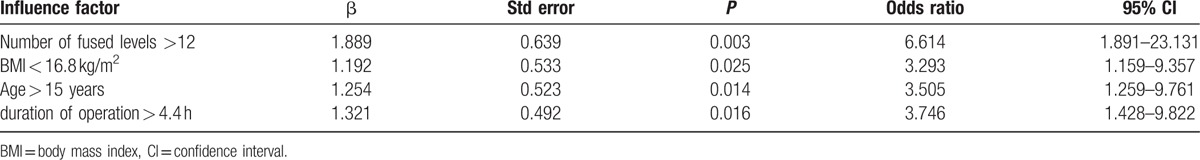
Independent influence factor of intraoperative massive blood loss.

### 3.3 Prognostic impact associated with massive blood loss

To maintain the steadiness of intraoperative hemodynamics, patients with massive blood loss (EBL/TBV ≥ 30%) received more intraoperative colloid infusion (*P* = 0.002) and blood transfusions (autologous and allogeneic red blood cells and fresh frozen plasma, *P* = 0.049, *P* < 0.001, and *P* < 0.001, respectively), as well as postoperative drainage volume (*P* = 0.001). The information is shown in Table 3.

**Table 3:**
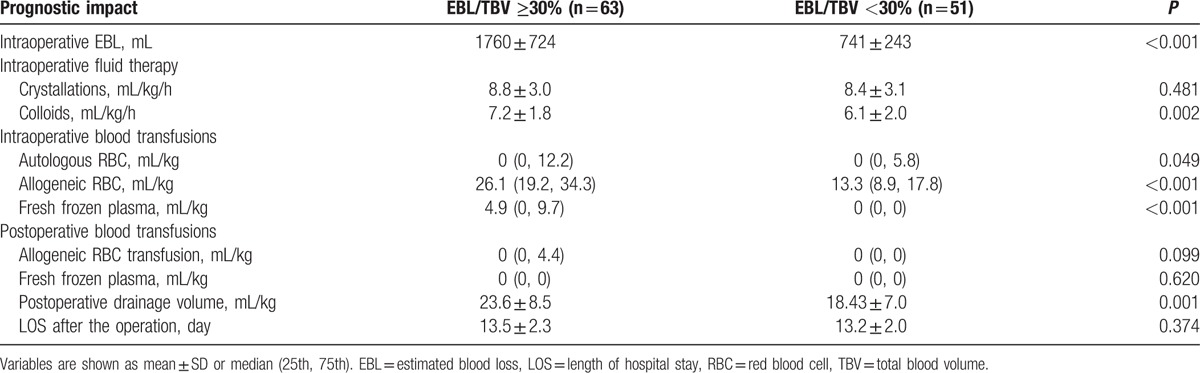
Prognostic impact associated with intraoperative massive blood loss.

## 4 Discussion

Compared to other types of scoliosis, NMS may be predisposed to higher morbidity associated with blood loss and the following therapy of blood product transfusion during the posterior spinal fusion operation.^[15–17]^ The phenomenon may be affected in a complicated manner by many factors including subclinical coagulation function abnormality,^[18]^ treatment with antiepileptic medications,^[19]^ reduced osteopenic bone and venous tone,^[15]^ malnutrition, and so on.

If the risk factors of massive blood loss could be better predicted before the operation, more appropriate blood conservation strategies could be used in these patients, which could reduce the demand of allogeneic blood transfusion.^[20–22]^ The disclosure of these risk factors may be useful for surgeons and anesthesiologists to make good preparations for massive blood loss and reduce the complications associated with transfusion.

According to the analyses of binary logistic regression, there were several risk factors of massive blood loss for NMS patients. First of all, the extent of fusion levels is an influencing factor of massive blood loss, and this factor is consistent with previous studies.^[4,23,24]^ More fusion levels mean more muscle and soft tissue stripping from vertebrae. Although the use of absorbable gelatin sponge and techniques such as firm packing and electrocautery could minimize intraoperative blood loss that derived from the surfaces of muscle and from vertebrae bleeding, the extent of fusion levels had a positive correlation with blood loss (Pearson correlation *r* value = 0.314, *P* = 0.001, data not listed). Also, more fusion levels increased the duration of the operation, which means that the exposure time of the muscle and vertebrae was extended. According to the regression equation, we can learn that the risk of massive blood loss increased 6.614 times when the number of fused levels was more than 12.

Another risk factor is lower BMI. As reported by Jain et al,^[25]^ the patients with smaller body sizes are almost always combined with worse nutritional status and greater osteopenia, which leads directly or indirectly to more blood loss during the operation. When the BMI was lower than 16.8 kg/m^2^, the risk of massive blood loss increased by 3.293 times.

The last 2 risk factors are, respectively, increasing age and prolonged duration of operation. As young patients grow up, they may be predisposed to a more rigid curve, and lower curve flexibility may be related to blood loss.^[26]^ During our study, the risk of massive blood loss increased 3.505 times with patients older than 15 years. Duration of operation had a positive correlation with blood loss (Pearson correlation *r* value = 0.602, *P* < 0.001, data not listed). The equation of binary logistic regression manifested that prolonged duration of operation increased the risk of massive blood loss by 3.746 times.

Besides, osteotomy procedure and preoperative Cobb angle lost statistical significance during the binary logistic regression. All the osteotomy procedures were Smith–Petersen osteotomy (SPO), bleeding less than other osteotomy types,^[27–29]^ which is probably why osteotomy lost statistical significance during the analyses of stepwise regression. As reported by previous studies,^[4,23,30]^ larger preoperative Cobb angle increased operation difficulty, and for this reason, it has long been known as a risk factor of massive blood loss. However, our study showed that the effect of a larger Cobb angle is not as much as the aforementioned risk factors.

Several advantages in this study should be mentioned. Primarily, this was a single-site investigation, and surgical technique and evaluation criteria of inspection were relatively uniform. Moreover, the target population of our study was the patient with NMS, and the age bracket was from 11 to 18; other types of scoliosis were excluded. Therefore, compared to previous studies, its internal validity is fairly high. Finally, compared with absolute blood loss, in the regard of expressed information of blood loss, EBL/TBV would be more convincing because it took into account the patient's weight and height.

There are several limitations that need to be disclosed. First of all, the volume of blood loss was quantified by the anesthesiologist as the difference between suction and irrigation volumes and the sum of gauze weight. As a result, the EBL was common with visual estimation and remained subjective. Furthermore, during this uncontrolled study, all the data were collected in a retrospective manner. Finally, all cases of NMS were derived from our medical institution. The nature of a single-center study is known to all, that is, its external validity is limited. For this reason, more future multiple-center researches are needed to identify the risk factors of massive blood loss.

The greatest contribution of this study is the conclusion that more than 12 fusion levels, ages older than 15 years, BMI less than 16.8 kg/m^2^, and duration of operation more than 4.4 hours were the risk factors of massive blood loss during the PSIF. These conclusions may be beneficial to anesthesiologists and surgeons in identifying patients with risk factors of massive blood loss during the perioperative period.
